# Temporal trends in cardiovascular health among Chinese urban children and adolescents, 2004–2019 pre-pandemic COVID-19

**DOI:** 10.3389/fpubh.2022.1023717

**Published:** 2022-10-14

**Authors:** Pei Xiao, Hong Cheng, Yinkun Yan, Dongqing Hou, Hongbo Dong, Xiaoyuan Zhao, Jie Mi

**Affiliations:** ^1^Center for Non-communicable Disease Management, Beijing Children's Hospital, Capital Medical University, National Center for Children's Health, Beijing, China; ^2^Department of Epidemiology, Capital Institute of Pediatrics, Beijing, China; ^3^Child Health Big Data Research Center, Capital Institute of Pediatrics, Beijing, China

**Keywords:** cardiovascular health, children and adolescents, risk factor, time trend analyses, COVID-19

## Abstract

**Objective:**

Little is known about pre-pandemic cardiovascular health (CVH) status and its temporal variation in Chinese children. Thus, we aimed to evaluate the secular trends and associated factors of CVH in Chinese urban children from 2004 to 2019.

**Methods:**

We identified 32,586 individuals in Beijing, aged 6 to 18 years, from three independent cross-sectional studies conducted in 2004, 2014, and 2019, respectively. CVH was assessed by 7 metrics according to modified American Heart Association criteria, including smoking, physical activity, diet, body mass index, total cholesterol, blood pressure, and fasting glucose. Multivariable logistic regression was used to assess the associations between sociodemographic characteristics and the ideal CVH status.

**Results:**

The proportion of ideal CVH decreased from 27.7% (boys 26.6%, girls 28.9%) in 2004 to 4.2% (boys 3.8%, girls 4.8%) in 2014, and then increased to 16.2% (boys 13.5%, girls 18.9%) in 2019. Overall, ideal smoking was the most prevalent CVH component during 2004–2019 (2004, 97.5%; 2014, 92.9%; 2019, 98.0%), while ideal physical activity (2004, 27.6%; 2014, 14.4%; 2019, 28.0%) and dietary intake (2004, 26.0%; 2014, 10.7%; 2019, 23.5%) were the least prevalent components. Notably, the proportion of ideal body mass index (2004, 77.5%; 2019, 59.7%) and blood pressure (2004, 73.6%; 2019, 67.3%) continuously decreased from 2004 to 2019. Girls, parental normal weight status, free of family CVD history, and lower levels in fat mass were associated with higher odds of ideal CVH.

**Conclusion:**

The cardiovascular health in Chinese urban children deteriorated during 2004–2019. Distinct strategies are required to mitigate socioeconomic inequity in the intervention of CVH promotion.

## Introduction

Cardiovascular disease (CVD), the largest contributor to global mortality, accounts for 40% of total deaths in China in 2016 (1, 2). Unlike the outstanding achievements in the reduction of CVD-related mortality made by developed countries, the burden of CVD continues to increase in China (3). Thus, several important policy documents have been issued by the Chinese government to set the goal of CVD prevention and control, including “Healthy China 2030,” “China's medium-to-long-term plan for the prevention and treatment of chronic diseases (2017–2025),” and “Healthy China Action (2019–2030)” (4). Importantly, growing life course epidemiological studies have highlighted the significant effects of early life CVD risk factors on CVD in adulthood, indicating that maintenance of cardiovascular health in childhood is warranted (3, 5).

To monitor cardiovascular health throughout the life course, the American Heart Association (AHA) offered a novel definition of cardiovascular health (CVH) metrics, consisting of 4 health behaviors (non-smoking, physical activity, body mass index [BMI], and diet) and 3 health factors (blood pressure [BP], total cholesterol [TC], and fasting blood glucose [FBG]) (6). So far, a limited number of studies have reported the CVH status before the past decade among Chinese children, and the trends in pediatric CVH status remain largely unknown (7–9). However, the status remains unclear after the implementation of health promotion policies, especially before the coronavirus disease 2019 (COVID-19) pandemic. Indeed, the COVID-19 pandemic has induced unhealthy lifestyles, which have a certain adverse effect on the CVH. Therefore, understanding the pre-pandemic CVH status among Chinese children will provide not only evidence for the evaluation of CVD control efforts but also the baseline data for the further assessments regarding the impact of COVID-19 on CVH. Besides, given the rapid socio-economic growth and urbanization in China, characterizing the temporal trends and demographic variation of CVH in children may better inform opportunities for earlier efficient prevention.

Here, leveraging data from three independent cross-sectional studies conducted in 2004, 2014, and 2019 pre-pandemic in Beijing, we sought to assess the temporal trend and its demographic disparities in CVH status among Chinese urban children.

## Materials and methods

### Study population

This study used the data from three population-based cross-section investigations conducted in Beijing, China. All the students in the selected schools were invited to participate in the investigations. Exclusion criteria included: inability to move (eg., deformity, fracture or prostheses); pregnancy; absence from school; and the inability to give informed consent. A total of 32 586 subjects with valid CVH metrics data were included in the current analysis. Each study was approved by the institutional review board of the Capital Institute of Pediatrics, and informed consent was obtained from all participants or their guardians (participants < 12 years).

#### Beijing child and adolescent metabolic syndrome (BCAMS) study: 2004

The BCAMS study is an ongoing cohort study to identify cardiovascular risk factors from childhood to adulthood. Baseline data were collected in 2004 on a representative sample of 19 539 Beijing children aged 6 to 18 years using a random cluster sampling method. In the present analysis, we included 18 739 subjects at baseline (96% of those eligible, 49% boys) who had valid information on CVH metric. Protocols for baseline measures have been described elsewhere (10).

#### China child and adolescent cardiovascular health (CCACH) study: 2014

The CCACH study was a school-based multicenter investigation of cardiovascular health among Chinese children in 2014. The standardized protocols have been described previously (11, 12). Briefly, a multistage sampling method was used to select a representative sample of children aged 6–18 years from 7 cities in China. To make data comparable, we included subjects from Beijing who had available CVH metrics data for the current analysis.

#### School-based cardiovascular and bone health promotion program (SCVBH): 2019

The SCVBH was a population-based cohort study initiated in November 2017. Its aim was to identify the risk factors for cardiovascular and bone health in school-aged children. A detailed description of the protocol and baseline survey results has been reported elsewhere (13, 14). Briefly, 15 391 students aged 6–16 years were enrolled using a stratified cluster sampling method at baseline in 2017. 13,837 subjects of them were then obtained in the follow-up investigation from November to December 2019 pre-pandemic. In the current analysis, we included 12 122 participants with valid CVH metrics data in the follow-up investigation.

### Data collection

Sociodemographic information (e.g., sex, age, household income, and educational level), lifestyle factors (e.g., smoking, drinking, physical activity, and dietary habits), and personal/family medical history (e.g., parental weight status) were collected by pretested and validated questionnaires (Supplementary methods). Household income was categorized into poor, middle, and high according to household annual income accounting for the year of assessment. Parental educational level was divided into below college vs. college or above. Parental weight status was defined as normal (< 24 kg/m^2^), overweight (24– < 28 kg/m^2^), and obesity (≥ 28 kg/m^2^). Parental history of cardiovascular disease was defined based on self-reported history of coronary artery disease and stroke. Passive smoking was defined as staying in a smoking environment for more than 1 day/week or whose parents were current smokers. Sedentary behavior was measured according to self-reported sedentary time and defined as ≥2 h/day by the Physical Activity Guidelines for Chinese (15). Sleep duration was calculated from self-reported sleep timings and individuals that slept 9 to 12 h/day (6–12 years) or 8 to 10 h/day (13–18 years) were considered to have an adequate sleep duration (16). Birth status was classified as premature (< 37 completed weeks of gestation) or not. Sexual maturity was estimated based on the occurrence of spermatorrhea or menstruation.

Physical examinations including anthropometric measures and body composition assessment were obtained following standardized protocols. BMI was calculated as weight (kg) divided by height (m^2^). Resting BP was measured from the right brachial artery three times with 1–2 min intervals in the morning, and the average of the last two readings was used for analysis. The body fat mass was measured using bioelectrical impedance analysis (BCAMS and SCVBH) and dual-energy X-ray absorptiometry (CCACH). The body fat mass percentage (FMP) was calculated by dividing the fat mass (grams) by total body weight (grams) and categorized into three groups by tertiles.

Fasting venous blood samples of participants in the CCACH and SCVBH were collected at the time of recruitment and then stored at −80°C in the central clinical laboratory until analysis. TC and FBG were measured by Olympus AU640 automatic chemistry analyzer (Olympus, Tokyo, Japan) in 2014 and Hitachi 7,080 biochemistry autoanalyzer (Hitachi, Tokyo, Japan) in 2019. For BCAMS in 2004, TC and FBG were determined in finger capillary blood by a testing device (Accutrend GCT, Roche Diagnostics, Mannheim, Germany).

### Cardiovascular health metrics

Due to the ethnic health disparities, we used modified AHA criteria to assess CVH in Chinses children and adolescents (6–9). The CVH metrics included 7 measures which can be split into 4 health behaviors (non-smoking, BMI, physical activity, and dietary patterns) and 3 health factors (BP, TC, and FBG). Specific definitions of poor, intermediate, and ideal CVH metrics for children were detailed in Table 1. The total CVH score was derived by the summation of the total number of ideal CVH metrics, ranging from 0 to 7. We defined the CVH as ideal if a participant attained a score of 6–7. Those with 3–5 and 0–2 ideal CVH metrics were described as having intermediate and poor CVH, respectively. Individuals who attained 3–4 in CVH behavior score were defined as ideal CVH behaviors, and 3 in CVH factor score as ideal CVH factors.

**Table 1:** Poor, intermediate, and ideal definitions of cardiovascular health metrics in Chinese children and adolescents using a modified American Heart Association recommendation.

**Metric**	**Poor**	**Intermediate**	**Ideal**
Smoking status	Tried prior 30 days	–	Never tried; never smoked a whole cigarette
BMI ^a^	>95th percentile	85th−95th percentile	< 85th percentile
Physical activity	None	0–59 min/d moderate or vigorous activity	≥60 min/d of moderate– or vigorous– intensity activity
Healthy diet pattern ^b^	0–1 components	2–3 components	4–5 components
Total cholesterol ^c^	≥200 mg/dL	170–199 mg/dL	< 170 mg/dL
Blood pressure ^c, d^	>95th percentile	90–95th percentile or SBP ≥120 or DBP ≥80 mm Hg	< 90th percentile
Fasting blood glucose^c^	≥126 mg/dL	100–125 mg/dL	< 100 mg/dL

BMI, body mass index; SBP, systolic blood pressure; DBP, diastolic blood pressure.

^a^Sex– and age–specific BMI percentiles for children and adolescents according to the Chinese reference standards.

^b^Five healthy diet components: fruits and vegetables ≥1/d, aquatic foods ≥1/week, bean–curd or dairy products ≥1/d, fried food or western fast food ≤ 1/week, sugar–sweetened beverages ≤ 1/week.

^c^Untreated values.

^d^Sex–, age–, and height–specific blood pressure percentiles for children and adolescents according to the Chinese reference standards.

Smoking exposure was evaluated by a self-report question as never tried (ideal) or tried in the past month (poor). BMI categories were defined as ideal (< 85th), intermediate (85th-95th), and poor (>95th) based on the sex- and age-specific BMI percentiles for Chinese children (17). Physical activity was assessed by the questions of frequency and duration of vigorous or moderate activity. Ideal physical activity was defined as moderate or vigorous activity ≥60 min/d as AHA recommended (6). Healthy dietary patterns were assessed by the following 5 components: (1) fruits and vegetables (≥1 time/day). (2) aquatic foods (≥1 time/week). (3) fried food or western fast food ( ≤ 1 time/week). (4) sugar-sweetened beverage (< 1 time/week); and (5) bean-curd or dairy products (≥1 time/day) (8). Subjects who achieved 4–5, 2–3, and 0–1 healthy dietary components were classified into the ideal, intermediate, and poor, respectively. Untreated BP levels were classified as ideal (< 90th), intermediate (90–95th), or poor (>95th) according to the age-, gender-, and height-specific blood pressure reference standards for Chinese children and adolescents (18). A FBG level of < 100, 100–125, and ≥126 mg/dL was set as the ideal, intermediate, and poor for the pediatric population, respectively (6). The TC concentrations were classified as ideal (< 170 mg/dL), intermediate (170–199 mg/dL), and poor (≥200 mg/dL) according to the AHA recommendation (6).

### Statistical analysis

Characteristics of participants were presented as mean (SD) for continuous variables and N (%) for categorical variables, and the differences in them across surveys were examined using analysis of variance or chi-squared tests. Mean (SD) of CVH score and proportions (95% *CI*) of each CVH metric status were performed by years in the total population and subgroups of sex and age. The Cochran-Armitage trend test was used to evaluate the significance of trends in ideal CVH metric prevalence over time. Multivariable logistic regression analyses were used to investigate the association between demographic factors and ideal CVH status. In addition, we calculated the population attributable risk (PAR) of overweight/obesity for each CVH factor from the logistic regression model by using *AF* package (version 0.1.5). For survey questions, any response of “refused” or “don't know” was assigned to a missing value. Multiple imputation with chained equations was performed for participants with missing covariate data, assuming data were conditionally missing at random. A total of 30 data sets were imputed using *mice* package (version 3.6.0), and regression coefficients were pooled using Rubin's rules. Stratified analyses were also performed according to survey year and sex. And sensitivity analyses were performed by including only complete cases to produce comparable conclusions.

A 2-tailed *P* ≤ 0.05 was considered statistically significant, and all analyses were performed by *R* software (version 3.4.0, www.cran.r-project.org).

## Results

### Characteristics of study population

A total of 32 586 participants aged 6–18 years with complete CVH metrics data were included in the present study. The mean (SD) age at participation was 12.3 (3.6) years, and 50% (95% *CI*: 49–51%) were boys. Among them, 10 473 (32%) individuals had missing data on covariates. The characteristics of participants by year are described in Table 2. In 2019 and 2014, parental educational level was significantly higher than that in 2004 (*P* < 0.001), but the parents were more likely to have overweight/obesity and a history of CVD (*P* < 0.001). Concerning children, BMI and systolic BP increased over time, while diastolic BP decreased.

**Table 2:** Characteristics of participants by survey year.

**Characteristics**	**Year**			***P***
	**2004**	**2014**	**2019**	
N	18739	1725	12122	
Boys, *N (%)*	9285 (49.5)	954 (55.3)	6025 (49.7)	< 0.001
Age, year (SD)	12.59 (3.30)	11.64 (3.70)	13.24 (3.30)	< 0.001
Household income, *N*(%)^a^				< 0.001
Poor	980 (5.8)	34 (3.3)	547 (5.0)	
Middle	3606 (21.5)	163 (15.7)	4281 (39.2)	
High	12194 (72.7)	839 (81.0)	6085 (55.8)	
Paternal educational level, *N*(%)^a^				< 0.001
Less than college	12949 (70.4)	255 (15.2)	5032 (44.2)	
College and above	5442 (29.6)	1420 (84.8)	6355 (55.8)	
Maternal educational level, *N*(%)^a^				< 0.001
Less than college	13140 (71.2)	329 (20.7)	4638 (40.8)	
College and above	5307 (28.8)	1260 (79.3)	6738 (59.2)	
Paternal weight status, *N*(%)^a^				< 0.001
Normal	8679 (48.2)	575 (36.4)	3594 (31.4)	
Overweight	7385 (41.0)	785 (49.7)	5022 (43.8)	
Obesity	1943 (10.8)	218 (13.8)	2844 (24.8)	
Maternal weight status, *N*(%)^a^				< 0.001
Normal	11849 (65.5)	1162 (73.0)	7042 (61.0)	
Overweight	4967 (27.5)	350 (22.0)	2951 (25.6)	
Obesity	1272 (7.0)	79 (5.0)	1542 (13.4)	
Parental cardiovascular disease history, *N*(%)^a^	2568 (13.7)	345 (21.4)	3034 (26.7)	< 0.001
Passive smoking, *N*(%)^a^	12496 (66.7)	772 (48.0)	3871 (32.8)	< 0.001
Sedentary time < 2 h, *N*(%)^a^	10408 (55.6)	1058 (72.9)	8386 (92.8)	< 0.001
Adequate sleep duration, *N*(%)^a^	12800 (69.4)	1249 (76.7)	6508 (55.4)	< 0.001
Premature, *N*(%)^a^	765 (4.6)	76 (4.4)	631 (5.7)	< 0.001
Sexual maturity, *N (%)*	8069 (43.1)	758 (43.9)	6053 (49.9)	< 0.001
Fat mass percentage, % (SD)^a^	19.6 (7.4)	29.8 (6.9)	25.7 (9.3)	< 0.001
Body mass index, kg/m^2^ (SD)	19.0 (4.0)	19.8 (4.4)	21.2 (4.9)	< 0.001
Systolic blood pressure, mmHg (SD)	104.0 (11.9)	110.1 (12.5)	112.4 (11.9)	< 0.001
Diastolic blood pressure, mmHg (SD)	66.5 (8.6)	63.7 (7.6)	62.1 (7.6)	< 0.001
Total cholesterol, mmol/L (SD)	4.00 (0.35)	4.23 (0.71)	4.03 (0.75)	< 0.001
Fasting glucose, mmol/L (SD)	4.62 (0.52)	5.60 (0.54)	5.14 (0.41)	< 0.001

^a^The number of missing values for household income, paternal educational level, maternal educational level, paternal weight status, maternal weight status, parental cardiovascular disease history, passive smoking, sedentary time, adequate sleep duration, premature, and fat mass percentage were 3857, 1133, 1174, 1541, 1372, 878, 433, 3382, 765, 2936, and 685, respectively.

### Trends in cardiovascular health metrics

The mean (SD) of CVH score and prevalence (95% *CI*) of ideal CVH in the total population and subgroups of sex and age are shown in Table 3. Overall, the CVH score decreased from 4.91 (1.01) in 2004 to 3.62 (1.17) in 2014 and then improved to 4.36 (1.17) in 2019 (*P* trend < 0.001). The prevalence of ideal CVH in 2019 was 16.2% (15.5–16.9%), which was higher than that (4.2%, 3.3–5.3%) in 2014 but still lower than that (27.7%, 27.1–28.4%) in 2004. Similar trends were found in the prevalence of ideal CVH behaviors and factors. In 2019, less than one-third (29.2%, 28.3–30.0%) and less than half of children (43.2%, 42.3–44.1%) attained ideal CVH behaviors and factors, respectively. From 2004 to 2019, girls had higher CVH scores and prevalence of ideal status than boys. Younger children were more likely to have ideal CVH in 2004, while no significant differences were found between age groups in 2014 and 2019.

**Table 3:** Cardiovascular health score and prevalence of ideal cardiovascular health in total population and subgroups of sex and age: 2004–2019.

**Population/year**	**N**	**Score**			**Ideal status %**		
		**CVH**	**CVH behaviors**	**CVH factors**	**Ideal CVH**	**Ideal CVH behaviors**	**Ideal CVH factors**
Overall							
2004	18739	4.91 (1.01)	2.29 (0.76)	2.62 (0.56)	27.7 (27.1–28.4)	37.2 (36.5–37.9)	66.1 (65.5–66.8)
2014	1725	3.62 (1.17)	1.80 (0.71)	1.82 (0.88)	4.2 (3.3–5.3)	14.0 (12.4–15.7)	24.9 (22.8–27.0)
2019	12122	4.36 (1.17)	2.09 (0.81)	2.27 (0.74)	16.2 (15.5–16.9)	29.2 (28.3–30.0)	43.2 (42.3–44.1)
*P* trend		< 0.001	< 0.001	< 0.001	< 0.001	< 0.001	< 0.001
Boys							
2004	9285	4.83 (1.04)	2.25 (0.79)	2.59 (0.57)	26.6 (25.7–27.5)	36.6 (35.6–37.6)	63.2 (62.2–64.2)
2014	954	3.43 (1.18)	1.73 (0.77)	1.70 (0.87)	3.8 (2.7–5.2)	14.8 (12.6–17.2)	19.4 (16.9–22.0)
2019	6025	4.19 (1.20)	2.02 (0.84)	2.17 (0.75)	13.5 (12.6–14.3)	27.0 (25.9–28.1)	36.3 (35.1–37.5)
*P* trend		< 0.001	< 0.001	< 0.001	< 0.001	< 0.001	< 0.001
Girls							
2004	9454	4.98 (0.97)	2.32 (0.73)	2.65 (0.55)	28.9 (28.0–29.8)	37.7 (36.7–38.7)	69.0 (68.1–70.0)
2014	771	3.87 (1.11)	1.90 (0.63)	1.97 (0.87)	4.8 (3.4–6.6)	13.0 (10.7–15.5)	31.6 (28.4–35.1)
2019	6097	4.54 (1.11)	2.16 (0.78)	2.38 (0.71)	18.9 (17.9–19.9)	31.3 (30.1–32.5)	50.1 (48.8–51.3)
*P* trend		< 0.001	< 0.001	< 0.001	< 0.001	< 0.001	< 0.001
6–11 years							
2004	8411	4.93 (1.01)	2.30 (0.77)	2.63 (0.56)	29.2 (28.2–30.2)	38.5 (37.5–39.5)	66.8 (65.8–67.8)
2014	933	3.63 (1.15)	1.85 (0.68)	1.79 (0.86)	4.3 (3.1–5.8)	14.1 (12.0–16.6)	22.5 (19.9–25.3)
2019	5767	4.36 (1.16)	2.11 (0.82)	2.26 (0.74)	15.7 (14.8–16.7)	29.9 (28.7–31.1)	42.3 (41.0–43.5)
*P* trend		< 0.001	< 0.001	< 0.001	< 0.001	< 0.001	< 0.001
12–18 years							
2004	10328	4.89 (1.00)	2.27 (0.75)	2.62 (0.56)	26.5 (25.7–27.4)	36.1 (35.2–37.0)	65.6 (64.7–66.5)
2014	792	3.61 (1.19)	1.75 (0.74)	1.86 (0.91)	4.2 (2.9–5.8)	13.8 (11.4–16.4)	27.7 (24.6–30.9)
2019	6355	4.36 (1.18)	2.08 (0.81)	2.29 (0.73)	16.6 (15.7–17.6)	28.5 (27.4–29.6)	44.1 (42.8–45.3)
*P* trend		< 0.001	< 0.001	< 0.001	< 0.001	< 0.001	< 0.001

**Figure 1:**
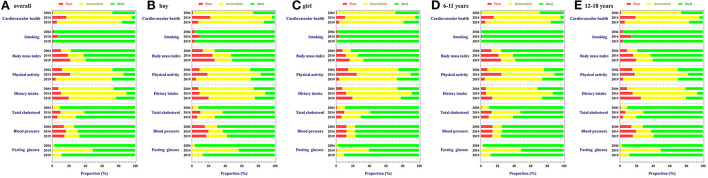
Trends in poor, intermediate, and ideal CVH metrics among children and adolescents in Beijing: 2004 to 2019. **(A–E)** Stand for proportions of CVH metrics status in overall population, boys, girls, children aged 6–12 years, and children aged 12–18 years, respectively.

The trends in proportion (95% *CI*) of poor, intermediate, and ideal CVH metrics among the total, sex-, and age-specific population are detailed in Figure 1 and Tables 4–6. Overall, the BMI and BP status among Chinese children steadily deteriorated from 2004 to 2019. The proportions of ideal TC, FBG levels, and dietary intake were higher in 2019 than that in 2014, even though still lower than that in 2004. Besides, the highest proportions of ideal smoking (98.0%, 97.7–98.2%) and physical activity (28.0%, 27.2–28.8%) status were reported in 2019 among children. The subgroup analyses showed that girls seemed to have better condition in most CVH metrics than boys except for physical activity and TC. In addition, children aged 6–11 years tended to have ideal smoking, dietary intake, and BP levels, whereas those aged 12–18 years had better status of BMI, physical activity, and TC levels.

**Table 4:** Proportions (95%*CI*) of poor, intermediate, and ideal cardiovascular metrics in total population: 2004–2019.

**CVH metrics**	**2004**	**2014**	**2019**	***P***
	**(*N* = 18,739)**	**(*N* = 1,725)**	**(*N* = 12,122)**	
CVH				< 0.001
Poor	1.4 (1.2–1.6)	17.2 (15.4–19.0)	5.8 (5.4–6.3)	
Intermediate	70.9 (70.2–71.5)	78.6 (76.6–80.5)	78.0 (77.2–78.7)	
Ideal	27.7 (27.1–28.4)	4.2 (3.3–5.3)	16.2 (15.5–16.9)	
Smoking				< 0.001
Poor	2.5 (2.3–2.7)	7.1 (5.9–8.4)	2.0 (1.8–2.3)	
Ideal	97.5 (97.3–97.7)	92.9 (91.6–94.1)	98.0 (97.7–98.2)	
Body mass index				< 0.001
Poor	10.6 (10.2–11.0)	19.0 (17.2–20.9)	21.9 (21.2–22.6)	
Intermediate	11.9 (11.5–12.4)	18.7 (16.9–20.6)	18.4 (17.8–19.1)	
Ideal	77.5 (76.9–78.1)	62.3 (60.0–64.6)	59.7 (58.8–60.5)	
Physical activity				< 0.001
Poor	12.1 (11.6–12.5)	21.6 (19.6–23.6)	4.4 (4.0–4.8)	
Intermediate	60.4 (59.7–61.1)	64.0 (61.7–66.3)	67.6 (66.8–68.4)	
Ideal	27.6 (26.9–28.2)	14.4 (12.8–16.2)	28.0 (27.2–28.8)	
Dietary intake				< 0.001
Poor	7.7 (7.3–8.0)	10.7 (9.2–12.2)	20.0 (19.3–20.7)	
Intermediate	66.4 (65.7–67.0)	78.6 (76.6–80.5)	56.6 (55.7–57.5)	
Ideal	26.0 (25.4–26.6)	10.7 (9.3–12.3)	23.5 (22.7–24.2)	
Total cholesterol				< 0.001
Poor	1.2 (1.1–1.4)	10.2 (8.8–11.7)	6.6 (6.2–7.1)	
Intermediate	7.8 (7.4–8.2)	28.5 (26.4–30.7)	22.0 (21.3–22.8)	
Ideal	91.0 (90.6–91.4)	61.3 (58.9–63.6)	71.4 (70.6–72.2)	
Blood pressure				< 0.001
Poor	14.1 (13.6–14.6)	16.8 (15.1–18.7)	14.9 (14.3–15.6)	
Intermediate	12.2 (11.8–12.7)	13.9 (12.3–15.6)	17.8 (17.1–18.5)	
Ideal	73.6 (73.0–74.2)	69.3 (67.0–71.4)	67.3 (66.4–68.1)	
Fasting glucose				< 0.001
Poor	0.1 (0.1–0.2)	0.6 (0.3–1.1)	0.1 (0.0–0.1)	
Intermediate	2.3 (2.1–2.5)	48.1 (45.7–50.4)	11.2 (10.6–11.8)	
Ideal	97.6 (97.4–97.8)	51.3 (48.9–53.7)	88.7 (88.2–89.3)	

**Table 5:** Proportions (95%*CI*) of poor, intermediate, and ideal cardiovascular metrics by sex: 2004–2019.

**CVH metrics**	**Boy**			***P***	**Girl**			***P***
	**2004**	**2014**	**2019**		**2004**	**2014**	**2019**	
	**(*N* = 9,285)**	**(*N* = 954)**	**(*N* = 6,025)**		**(*N* = 9,454)**	**(*N* = 771)**	**(*N* = 6,097)**	
CVH				< 0.001				< 0.001
Poor	1.7 (1.4–2.0)	22.2 (19.6–25.0)	7.8 (7.2–8.5)		1.1 (0.9–1.3)	10.9 (8.8–13.3)	3.9 (3.4–4.4)	
Intermediate	71.8 (70.8–72.7)	74.0 (71.1–76.8)	78.7 (77.6–79.7)		70.0 (69.1–70.9)	84.3 (81.5–86.8)	77.2 (76.2–78.3)	
Ideal	26.6 (25.7–27.5)	3.8 (2.7–5.2)	13.5 (12.6–14.3)		28.9 (28.0–29.8)	4.8 (3.4–6.6)	18.9 (17.9–19.9)	
Smoking				< 0.001				< 0.001
Poor	3.7 (3.4–4.2)	9.6 (7.8–11.7)	3.5 (3.0–4.0)		1.3 (1.1–1.5)	3.9 (2.6–5.5)	0.6 (0.4–0.8)	
Ideal	96.3 (95.8–96.6)	90.4 (88.3–92.2)	96.5 (96–97)		98.7 (98.5–98.9)	96.1 (94.5–97.4)	99.4 (99.2–99.6)	
Body mass index				< 0.001				< 0.001
Poor	13.3 (12.6–14)	24.8 (22.1–27.7)	27.4 (26.2–28.5)		7.9 (7.4–8.5)	11.8 (9.6–14.3)	16.5 (15.6–17.5)	
Intermediate	14.1 (13.4–14.8)	22 (19.4–24.8)	20.8 (19.8–21.9)		9.8 (9.2–10.4)	14.5 (12.1–17.2)	16.1 (15.2–17.0)	
Ideal	72.6 (71.7–73.5)	53.1 (49.9–56.3)	51.8 (50.6–53.1)		82.3 (81.5–83)	73.7 (70.4–76.7)	67.4 (66.2–68.6)	
Physical activity				0.051				< 0.001
Poor	9.4 (8.8–10.0)	18.9 (16.4–21.5)	5.0 (4.5–5.6)		14.7 (14–15.4)	24.9 (21.9–28.1)	3.8 (3.3–4.3)	
Intermediate	60.6 (59.6–61.6)	63.6 (60.5–66.7)	65.9 (64.7–67.1)		60.2 (59.2–61.2)	64.5 (61–67.8)	69.3 (68.1–70.4)	
Ideal	30.0 (29.1–31.0)	17.5 (15.1–20.1)	29.1 (27.9–30.2)		25.1 (24.2–26.0)	10.6 (8.5–13.0)	26.9 (25.8–28.1)	
Dietary intake				< 0.001				< 0.001
Poor	7.9 (7.3–8.4)	9.4 (7.7–11.5)	20.2 (19.2–21.2)		7.5 (6.9–8)	12.2 (10–14.7)	19.8 (18.8–20.8)	
Intermediate	66.5 (65.5–67.4)	78.6 (75.9–81.2)	55.3 (54.1–56.6)		66.3 (65.3–67.2)	78.6 (75.5–81.4)	57.8 (56.5–59)	
Ideal	25.7 (24.8–26.6)	11.9 (10–14.2)	24.5 (23.4–25.6)		26.3 (25.4–27.2)	9.2 (7.3–11.5)	22.4 (21.4–23.5)	
Total cholesterol				< 0.001				< 0.001
Poor	1.1 (0.9–1.3)	10.4 (8.5–12.5)	6.5 (5.9–7.2)		1.4 (1.2–1.6)	10.0 (8–12.3)	6.7 (6.0–7.3)	
Intermediate	6.4 (5.9–7)	26.6 (23.8–29.6)	21.2 (20.2–22.3)		9.1 (8.5–9.7)	30.9 (27.6–34.3)	22.8 (21.8–23.9)	
Ideal	92.5 (91.9–93)	63.0 (59.8–66.1)	72.2 (71.1–73.3)		89.5 (88.9–90.1)	59.1 (55.6–62.6)	70.5 (69.4–71.7)	
Blood pressure				< 0.001				0.632
Poor	15.6 (14.9–16.4)	20.0 (17.5–22.7)	17.5 (16.5–18.5)		12.7 (12.0–13.3)	12.8 (10.6–15.4)	12.4 (11.6–13.3)	
Intermediate	14.6 (13.9–15.4)	17.2 (14.8–19.7)	24.7 (23.7–25.9)		9.9 (9.3–10.5)	9.9 (7.8–12.2)	11.0 (10.2–11.8)	
Ideal	69.7 (68.8–70.7)	62.8 (59.6–65.9)	57.8 (56.5–59.0)		77.4 (76.6–78.3)	77.3 (74.2–80.2)	76.6 (75.6–77.7)	
Fasting glucose				< 0.001				< 0.001
Poor	0.1 (0.0–0.2)	0.5 (0.2–1.2)	0.0 (0–0.1)		0.1 (0.1–0.2)	0.8 (0.3–1.7)	0.1 (0.0–0.2)	
Intermediate	3.2 (2.8–3.5)	55.6 (52.3–58.7)	12.9 (12.1–13.8)		1.5 (1.2–1.7)	38.8 (35.3–42.3)	9.5 (8.8–10.3)	
Ideal	96.7 (96.4–97.1)	43.9 (40.7–47.1)	87.1 (86.2–87.9)		98.4 (98.2–98.7)	60.4 (56.9–63.9)	90.4 (89.7–91.1)	

**Table 6:** Proportions (95%*CI*) of poor, intermediate, and ideal cardiovascular metrics by age: 2004–2019.

**CVH metrics**	**6–11 years**			***P***	**12–18 years**			***P***
	**2004** **(*N* = 8,411)**	**2014** **(*N* = 933)**	**2019** **(*N* = 5,767)**		**2004** **(*N* = 10,328)**	**2014** **(*N* = 792)**	**2019** ***(N* = 6,355)**	
CVH				< 0.001				< 0.001
Poor	1.2 (1.0–1.4)	15.8 (13.5–18.3)	5.7 (5.1–6.3)		1.6 (1.3–1.8)	18.8 (16.1–21.7)	6.0 (5.4–6.6)	
Intermediate	69.6 (68.7–70.6)	80 (77.2–82.5)	78.6 (77.5–79.6)		71.9 (71–72.7)	77 (73.9–79.9)	77.4 (76.4–78.4)	
Ideal	29.2 (28.2–30.2)	4.3 (3.1–5.8)	15.7 (14.8–16.7)		26.5 (25.7–27.4)	4.2 (2.9–5.8)	16.6 (15.7–17.6)	
Smoking				< 0.001				< 0.001
Poor	1.0 (0.8–1.2)	1.8 (1.1–2.9)	0.4 (0.3–0.6)		3.7 (3.4–4.1)	13.3 (11.0–15.8)	3.5 (3.1–4.0)	
Ideal	99.0 (98.8–99.2)	98.2 (97.1–98.9)	99.6 (99.4–99.7)		96.3 (95.9–96.6)	86.7 (84.2–89.0)	96.5 (96.0–96.9)	
Body mass index				< 0.001				< 0.001
Poor	13.0 (12.3–13.7)	21.5 (18.9–24.3)	24.5 (23.4–25.7)		8.6 (8.1–9.2)	16.0 (13.5–18.8)	19.5 (18.5–20.5)	
Intermediate	11.5 (10.8–12.2)	16.9 (14.6–19.5)	16.7 (15.8–17.7)		12.3 (11.7–12.9)	20.7 (17.9–23.7)	20.0 (19.0–21.0)	
Ideal	75.5 (74.6–76.4)	61.5 (58.3–64.7)	58.7 (57.5–60.0)		79.1 (78.3–79.9)	63.3 (59.8–66.6)	60.5 (59.3–61.7)	
Physical activity				< 0.001				< 0.001
Poor	8.3 (7.7–8.9)	24.9 (22.1–27.8)	4.9 (4.3–5.5)		15.2 (14.5–15.9)	17.7 (15.1–20.5)	3.9 (3.5–4.5)	
Intermediate	67.8 (66.8–68.8)	63.8 (60.6–66.9)	69.2 (68.0–70.4)		54.4 (53.4–55.3)	64.3 (60.8–67.6)	66.2 (65.0–67.3)	
Ideal	24.0 (23.1–24.9)	11.4 (9.4–13.6)	25.9 (24.8–27.1)		30.5 (29.6–31.4)	18.1 (15.4–20.9)	29.9 (28.8–31.0)	
Dietary intake				< 0.001				< 0.001
Poor	6.4 (5.9–7.0)	6.5 (5.0–8.3)	14.1 (13.3–15.1)		8.7 (8.1–9.2)	15.5 (13.1–18.2)	25.3 (24.2–26.4)	
Intermediate	61.9 (60.9–62.9)	79.8 (77.1–82.4)	59.4 (58.1–60.6)		70.0 (69.1–70.9)	77.1 (74.1–80.0)	54.0 (52.8–55.3)	
Ideal	31.7 (30.7–32.7)	13.6 (11.5–16)	26.5 (25.4–27.7)		21.3 (20.6–22.1)	7.3 (5.6–9.4)	20.7 (19.7–21.7)	
Total cholesterol				< 0.001				< 0.001
Poor	1.2 (1.0–1.5)	13.3 (11.2–15.6)	9.7 (9.0–10.5)		1.2 (1.0–1.5)	6.6 (4.9–8.5)	3.8 (3.3–4.3)	
Intermediate	8.4 (7.8–9.0)	34.4 (31.4–37.6)	28.9 (27.7–30.0)		7.3 (6.8–7.8)	21.6 (18.8–24.6)	15.8 (14.9–16.8)	
Ideal	90.3 (89.7–91.0)	52.3 (49.0–55.6)	61.4 (60.1–62.7)		91.5 (90.9–92)	71.8 (68.6–75)	80.4 (79.4–81.4)	
Blood pressure				0.326				< 0.001
Poor	14.4 (13.7–15.2)	14.5 (12.3–16.9)	14.0 (13.2–15.0)		13.9 (13.2–14.6)	19.6 (16.9–22.5)	15.7 (14.8–16.7)	
Intermediate	10.7 (10.0–11.4)	10.8 (8.9–13.0)	10.2 (9.4–11.0)		13.5 (12.9–14.2)	17.6 (15.0–20.4)	24.7 (23.7–25.8)	
Ideal	74.9 (73.9–75.8)	74.7 (71.8–77.5)	75.8 (74.6–76.9)		72.6 (71.7–73.4)	62.9 (59.4–66.3)	59.5 (58.3–60.7)	
Fasting glucose				< 0.001				< 0.001
Poor	0.0 (0.0–0.1)	0.5 (0.2–1.2)	0.0 (0.0–0.1)		0.1 (0.1–0.2)	0.8 (0.3–1.6)	0.1 (0.0–0.2)	
Intermediate	2.4 (2.1–2.7)	47.9 (44.7–51.2)	11.5 (10.6–12.3)		2.2 (2–2.6)	48.2 (44.7–51.8)	11.0 (10.2–11.8)	
Ideal	97.6 (97.2–97.9)	51.6 (48.3–54.8)	88.5 (87.7–89.3)		97.6 (97.3–97.9)	51.0 (47.5–54.5)	88.9 (88.1–89.7)	

**Figure 2:**
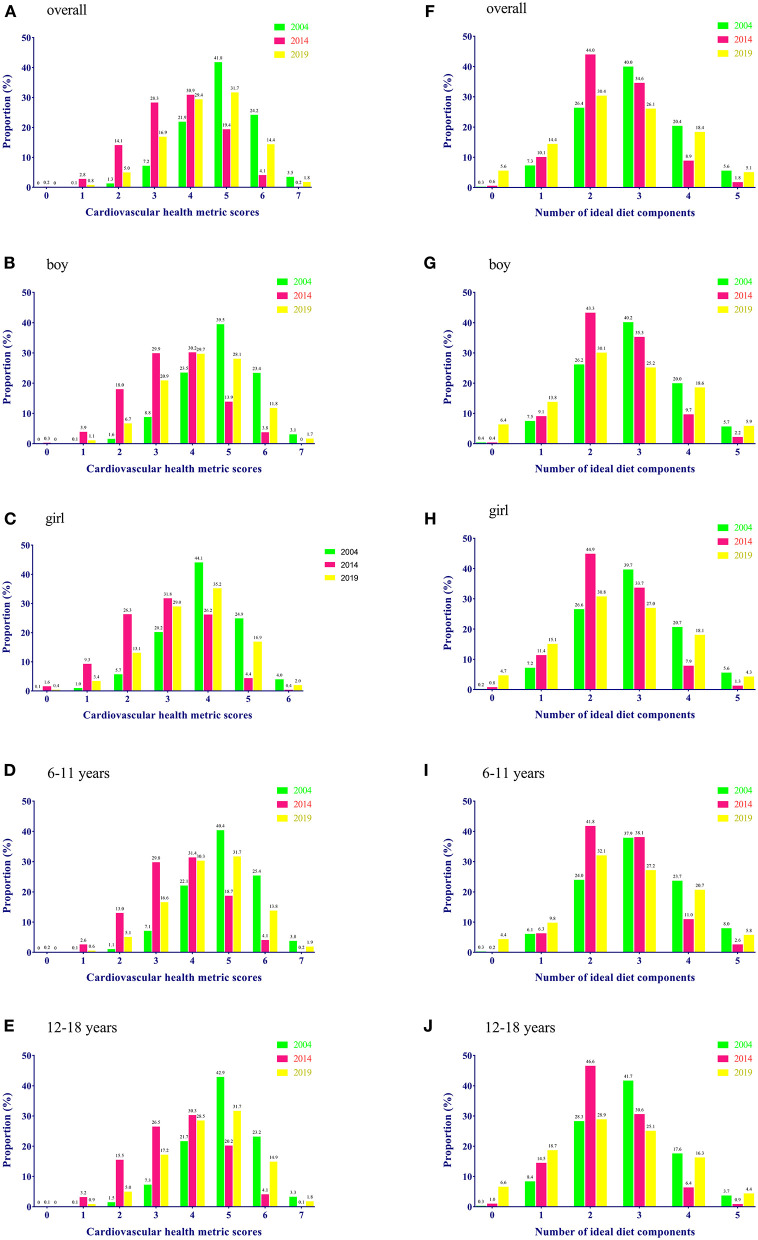
Distribution of CVH metrics score and healthy diet components in total population and subgroups of sex and age: 2004–2019. **(A–E)** Stand for the distribution of CVH metrics score in overall population, boys, girls, children aged 6–12 years, and children aged 12–18 years, respectively. **(F–J)** Stand for the number of ideal diet components in overall population, boys, girls, children aged 6–12 years, and children aged 12–18 years, respectively.

**Figure 3:**
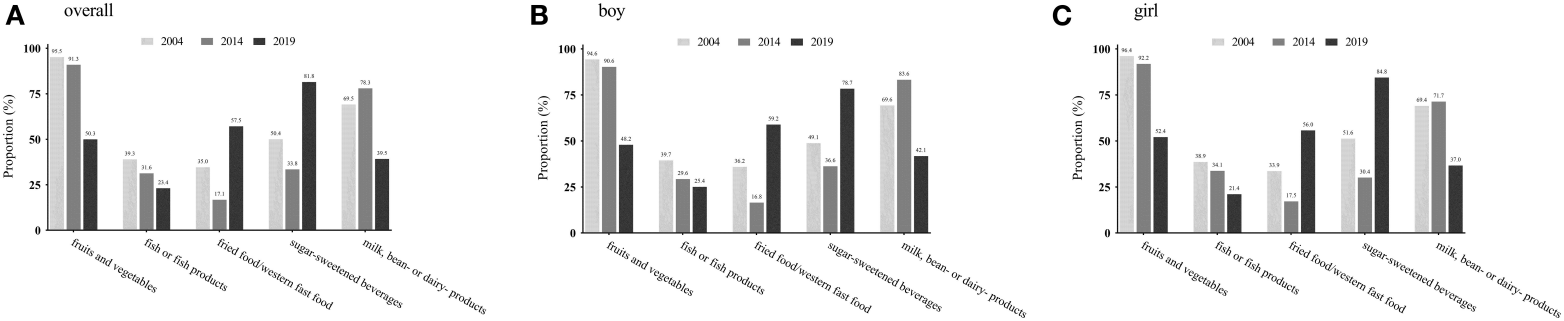
Prevalence of each ideal healthy diet component in total population and subgroups of sex: 2004–2019. **(A–C)** Stand for the prevalence of each ideal healthy diet component in overall population, boys, and girls, respectively.

The distribution of CVH metrics scores and the number of healthy diet components by sex and age groups from 2004 to 2019 are illustrated in Figure 2. A very low proportion (2004: 3.5%, 2014: 0.2%, and 2019: 1.8%) had all 7 ideal CVH metrics in the total population. The majority of children (87.9%) were with a score of 4–6 in CVH metrics in 2004, which reduced to 3–5 in 2014 (78.6%) and 2019 (78.0%). Notably, 5.9% of children reached none of the five ideal diet components in 2019, compared with only 0.3% in 2004 and 0.6% in 2014. Figure 3 presents the proportion of each ideal healthy diet component by year in the total and sex-specific population. In 2004 and 2014, more than 90% of children attained the ideal frequency of fruits and vegetables intake; however, it declined to 50% in 2019. Meanwhile, a growing number of children limited sugar-sweetened beverages and fried food/western fast food intake in 2019 compared to those in 2004–2014.

### Related factors of ideal cardiovascular health

**Figure 4:**
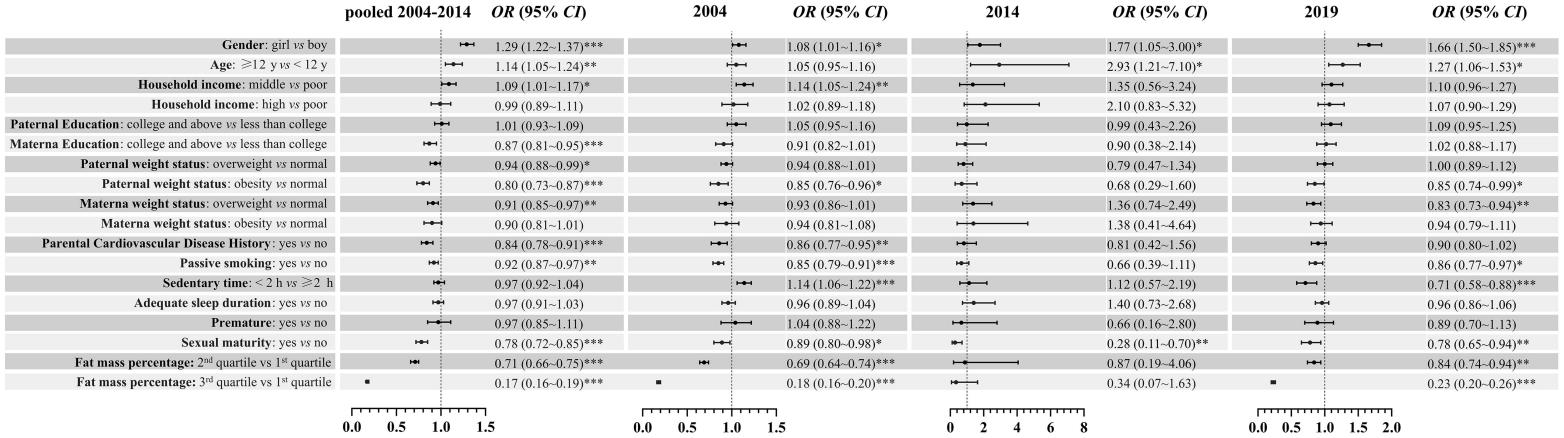
Association [OR (95%CI)] between demographic factors and ideal cardiovascular health among children and adolescents in Beijing: 2004–2019. *0.01 ≤ *P* < 0.05; **0.001 ≤ *P* < 0.01; ***P < 0.001.

**Figure 5:**
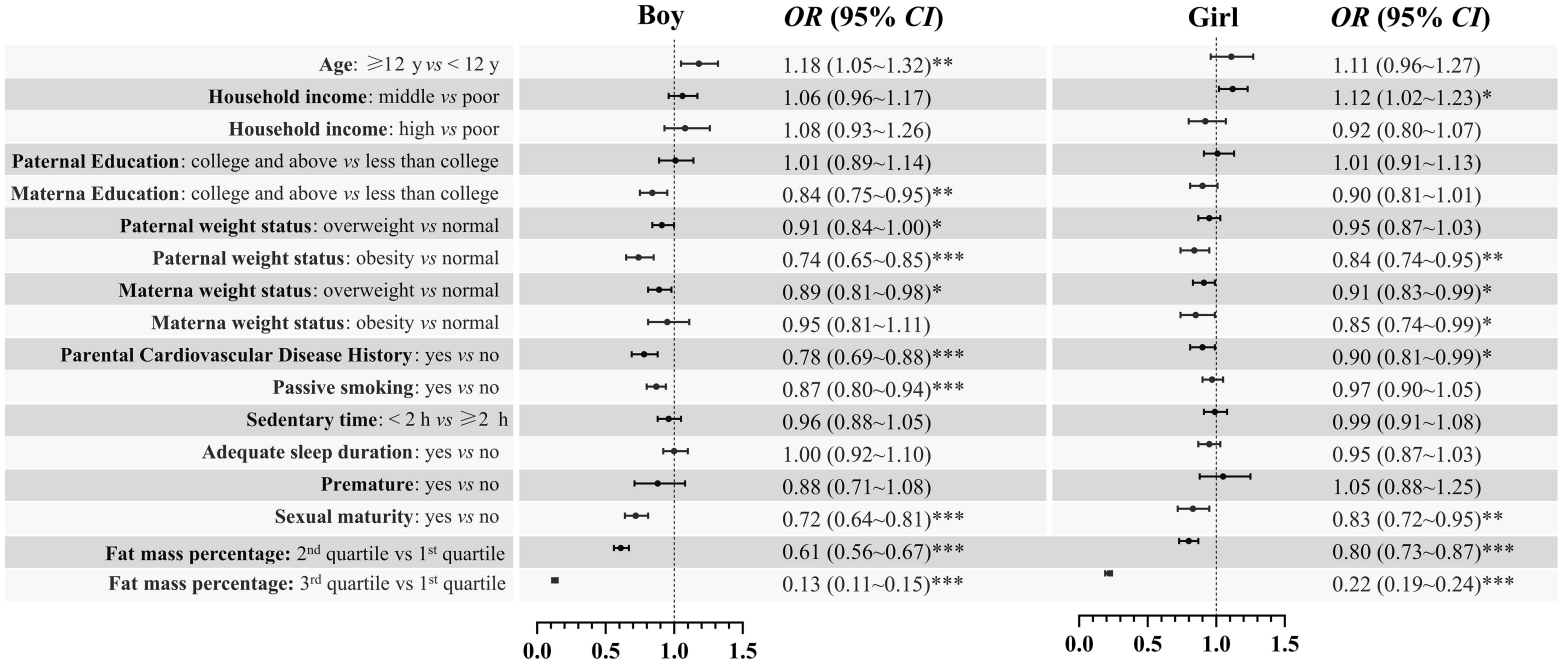
Association [OR (95%CI)] between demographic factors and ideal cardiovascular health among children and adolescents in Beijing by sex. *0.01 ≤ *P* < 0.05; **0.001 ≤ *P* < 0.01; ***P < 0.001.

**Figure 6:**
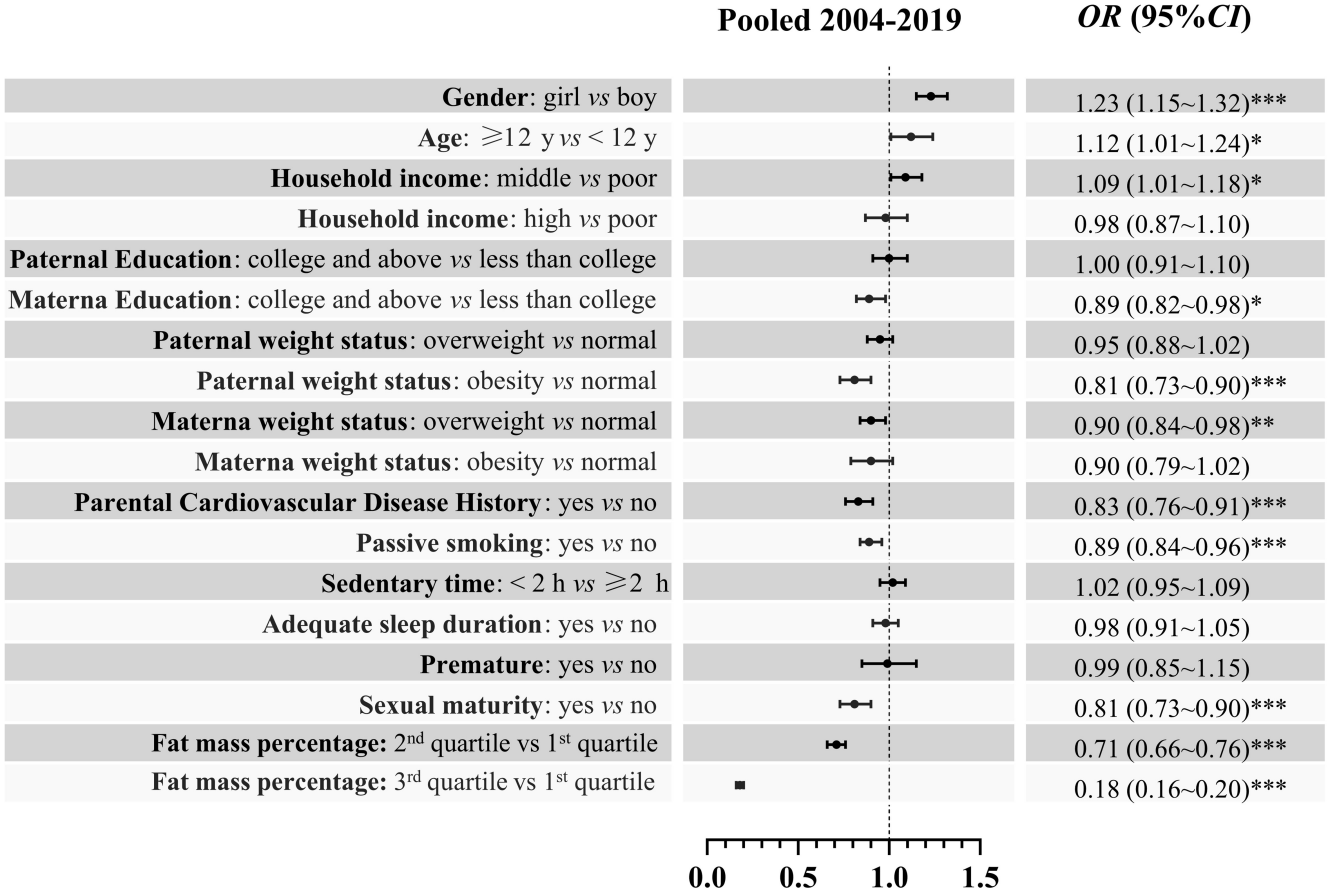
Sensitivity analyses with complete case: Association [OR (95%CI)] between demographic factors and ideal cardiovascular health among children and adolescents in Beijing. *0.01 ≤ *P* < 0.05; **0.001 ≤ *P* < 0.01; ***P < 0.001.

The associations between demographic factors and ideal CVH in the pooled data and each year are given in Figure 4. Generally, girls, older age (≥ 12 years), and middle household income were significantly associated with a higher prevalence of ideal CVH. Individuals with overweight/obese fathers, overweight mothers, parental history of CVD, passive smoking, and high fat mass percentage were significantly associated with decreased odds of ideal CVH compared to the reference group. In the subgroup analysis of sex, we found that age and passive smoking were associated with the prevalence of ideal CVH only in boys (Figure 5). A consistent result for the association between demographic factors and ideal CVH was given in the sensitivity analysis using complete cases without missing data (Figure 6).

## Discussion

To our knowledge, this is the first study to report pre-pandemic ideal CVH status among Chinese urban children from COVID-19 and present the trends and demographic variation in pediatric CVH status over the past two decades. Mainly, we observed a deteriorating trend in ideal CVH among Chinese urban children from 2004 (27.7%, 27.1–28.4%) to 2014 (4.2%, 3.3–5.3%). Nevertheless, the trend was estimated to be optimistic from 2014 to 2019 (16.2%, 15.5–16.9%). In addition, demographical variations were found in the distribution of CVH, including sex, age, parental weight status, parental history of CVD, and passive smoking, which may have important implications for developing CVD preventive strategies.

CVD, the largest single contributor to global mortality, was projected to cause >5 million premature deaths among men and 2.8 million among women worldwide by 2025 (2). Growing evidence indicates the pathogenic process of CVD begins early in life, highlighting the prevention of CVD should start from childhood (3). Thus, a pediatric companion focusing on cardiovascular health promotion and disease reduction throughout the life course was proposed by the AHA (6). Although most children were born with all cardiovascular health components, they experienced a decline in health factors and behaviors over time. Using the AHA recommended CVH metrics definition, we found that the majority of Chinese urban children had 4–6 ideal CVH metrics in 2004 while 3–5 in 2014 and 2019. Similarly, previous studies also reported that the distribution of ideal CVH metrics number among Chinese adults and children was concentrate between 3–5 in 2010, and only 0.2% of adults and 1.7% of children met all 7 ideal metrics (7, 19). According to our study population, the prevalence of meeting all 7 metrics ranged from 0.2 to 3.5% during the last two decades, suggesting primordial prevention should begin at an early age.

In the present study, ideal smoking status has been the most prevalent CVH component in all sex and age groups from 2004 to 2019, with the highest prevalence of 98% observed in 2019. Partially in line with our results, Zhu et al. found the prevalence of ideal smoking considering only active smoking was 98.7% among Chinese children; however, the prevalence was reduced by more than one-half when further accounting for passive smoking (7). With the Chinese government's efforts to ban smoking in public recently, we optimistically found the proportion of exposure to passive smoking declined from two-thirds in 2004 to one-third in 2019. Despite a much higher prevalence of ideal smoking status observed in Chinese children compared to the European and American pediatric population (20, 21), public health campaigns are still needed to consolidate the achievements of tobacco control.

It is now well established that diet pattern and physical activity are critical behavior factors for the progress of CVD. Unfortunately, consistent with previous reports (7–9), we found ideal diet and physical activity were the least prevalent among all ideal CVH metrics in Chinese urban children, with a prevalence of less than one-third. This worrying finding was consistent across all sex and age groups. Worse than Chinese children, < 0.5% of US children had an ideal diet pattern and approximately 91% were classified as having a poor diet pattern (22). Fish or fish product consumption, a protective factor of CVD (23), was with the least and decreased proportion of ideal status among our study population over time. In addition, the proportion of children who attained the ideal fruits and vegetables in 2019 has decreased by nearly half compared to 2004–2014. Optimistically, our results exhibited that the western/new affluence dietary patterns, including fried/western fast food and sugar-sweetened beverages, were less popular among Chinese urban children in 2019 compared to that in 2004–2014. As a developing country, China is experiencing an accelerating diet pattern transition due to socioeconomic development, and thus efforts are still needed to promote a healthy diet for children. Regarding physical activity, only < 30% of children engaged in ≥60 min of moderate- to vigorous-intensity physical activity per day. With the implementation of a series of health promotion policies issued by the Chinese government in the past 5 years, the proportion of children that met the ideal physical activity level largely increased in 2019 (28.0%) compared with 2014 (14.4%). However, in the circumstances of ongoing COVID-19 spread, governments have implemented school closures and encouraged students to take online classes as emergency measures, which may have a great impact on children's physical activity and sedentary behavior (24, 25), In a natural experimental longitudinal study, 435 min reduction on average time spent in PA and approximately 30 h per week increased in screen time were observed among children and adolescents in Shanghai during pandemic (24). Thus, further studies are still needed to assess the negative impact of the COVID-19 pandemic on CVH behavior health among Chinese urban children.

Obesity, demonstrated by many reports, tracks more strongly than any other major CVD risk factor from childhood to adulthood (26). As an easy-to-calculated CVH metric, BMI is widely recommended to screen weight status among children and adults. We observed a continuous decrease in the proportion of ideal BMI status among Chinese urban children during 2004–2019. Despite efforts over the last decade to prevent and control obesity, our results exhibited that the prevalence of obesity among children in 2019 (21.9%) has increased by nearly 2 times than that in 2004 (10.6%), with a worse situation in boys. Partially in line with our findings, Wang et al. estimated about half of adults and a fifth of children in China had overweight and obesity nowadays, making China the country with the highest number of obese individuals (27). Together with prior reports, our data indicated the previous efforts were clearly inadequate for overcoming the rapid epidemic of overweight and obesity in China. Furthermore, in parallel with the obesity epidemic, the BP levels in Chinese urban children were getting worse during 2004–2019, especially in boys. Our results showed that the PAR for hypertension due to overweight/obesity steadily increased from 31% in 2004 to 47% in 2019 (Table 7), which suggests that obesity remained the dominant determinant for hypertensive children. According to previous epidemiological surveys, BP levels and the prevalence of hypertension have been also increasing in US children and adolescents in the past years, explained partially by the rise in obesity rates (28). The other two CVH health factors in our study population, ideal TC and FBG, presented a similar trend that dramatically decreased from 2004 to 2014 and then rose in 2019. Ideal FBG was found as the most prevalent metric in previous studies of Chinese, American, and European children (7, 29, 30). Analogous to their findings, the proportion of ideal FBG was second only to ideal smoking status in this study.

**Table 7:** The change in PAR (%) for CVH factor due to overweight/obesity.

**CVH factor**	**PAR due to due to overweight/obesity (95%** ***CI*** **)**		
	**2004**	**2014**	**2019**
Poor blood pressure			
Overall	31.0 (28.8–33.3)***	40.5 (32.2–48.7)***	47.2 (43.8–50.5)***
Boy	36.2 (32.9–39.5)***	42.5 (31.2–53.9)***	49.3 (44.5–54.1)***
Girl	24.5 (21.4–27.5)***	32.5 (20.0–45.0)***	42.4 (37.5–47.2)***
Poor total cholesterol			
Overall	27.6 (19.4–35.8)***	6.5 (−4.6–17.6)	4.6 (−0.1–10.1)
Boy	43.5 (30.5–56.6)***	10.0 (−7.6–27.7)	9.3 (−0.1–18.5)
Girl	17.4 (7.7–27.2)***	2.1 (−11.0–15.2)	1.1 (−5.5–7.7)
Poor fasting blood glucose			
Overall	45.7 (17.0–74.3)**	56.2 (14.1–98.4)**	14.3 (10.1–18.5)***
Boy	49.7 (12.8–86.5)***	80.9 (79.6–82.2)***	17.1 (10.8–23.3)***
Girl	51.0 (14.1–87.9)**	54.7 (3.7–99.8)*	8.0 (2.5–13.6)**

PAR, population attributable risk; CVH, cardiovascular health; * 0.01 ≤ P < 0.05; ** 0.001 ≤ P < 0.01; ***P < 0.001.

Consistent with previous studies (7–9, 31–33), sex disparities in CVH among Chinese urban children were persistently observed over time. Relative to boys, the ideal CVH metrics of girls were better in smoking, BMI, BP, and FBG, whereas they were worse in physical activity and TC. Overall, middle household income, parental normal weight status, free of family CVD history and passive smoking, sexual immaturity, and lower levels in fat mass percentage were significantly associated with higher odds of ideal CVH status. Thus, distinct strategies are required to mitigate socioeconomic inequity in the intervention of CVH promotion.

The findings from the present study should be interpreted in light of its strengths and limitations. To our knowledge, this is the first study to report pre-COVID-19 CVH status in Chinese urban children, which provides the baseline data for assessing the impact of the pandemic on children's CVH. Besides, we further examined the trends and related factors of CVH from 2004 to 2019. This emphasizes a future challenge regarding CVD prevention. In an attempt to minimize the selection and information bias induced by the heterogeneity among three investigations, we used uniform variable definition and reported the CVH status according to sociodemographic characteristics. However, our study population was all drawn from urban areas, and thus might limit the generalizability to rural areas. And finger capillary blood collected in 2004 might slightly underestimate the fasting glucose levels. In addition, information collected by self-reports could induce a recall bias. Finally, the cross-sectional nature of the study cannot draw the causal inference.

In summary, despite signs of an improvement in cardiovascular health among Chinese urban children during 2014–2019, it is still worse than that in 2004. Notably, with the low prevalence of ideal physical activity and dietary intake, children's BMI and BP status have constantly deteriorated over time. The demographic disparities in children's CVH suggest that distinct strategies are needed for equitable CVH promotion.

## Data availability statement

The raw data supporting the conclusions of this article will be made available by the authors, without undue reservation.

## Ethics statement

The studies involving human participants were reviewed and approved by Institutional Review Board of the Capital Institute of Pediatrics. Written informed consent to participate in this study was provided by the participants' legal guardian/next of kin.

## Author contributions

JM: conceptualization, methodology, supervision, and writing—original draft. PX and HC: data curation, formal analysis, and writing—original draft preparation. PX, YY, and HD: visualization and software. HC, DH, and XZ: investigation, reviewing, and editing. JM and PX: had full access to all the data in the study and takes responsibility for the integrity of the data and the accuracy of the data analysis. All authors contributed to the article and approved the submitted version.

## Funding

The study was supported by the National Natural Science Foundation of China (81973110), the Beijing Municipal Natural Science Foundation (7214277), the National Key Research and Development Program of China (2016YFC0900602), and the Beijing Municipal Science and Technology Commission (H030930030130).

## Conflict of interest

The authors declare that the research was conducted in the absence of any commercial or financial relationships that could be construed as a potential conflict of interest.

## Publisher's note

All claims expressed in this article are solely those of the authors and do not necessarily represent those of their affiliated organizations, or those of the publisher, the editors and the reviewers. Any product that may be evaluated in this article, or claim that may be made by its manufacturer, is not guaranteed or endorsed by the publisher.

## Abbreviations

AHA: American Heart Association
BMI: body mass index
BP: blood pressure
BCAMS: Beijing Child and Adolescent Metabolic Syndrome Study
CCACH: China Child and Adolescent Cardiovascular Health Study
COVID-19: coronavirus disease 2019
CVD: cardiovascular disease
CVH: cardiovascular health
FBG: fasting blood glucose
FMP: fat mass percentage
PAR: population attributable risk
SCVBH: School-based Cardiovascular and Bone Health Promotion Program
TC: total cholesterol.
