# Supplementary material for: Temporal trends in cardiovascular health among Chinese urban children and adolescents, 2004–2019 pre-pandemic COVID-19
Source: Front Public Health. 2022 Oct 14;10:1023717. doi: 10.3389/fpubh.2022.1023717 (PMC9614081; doi:10.3389/fpubh.2022.1023717)
Supplement: Supplementary file 1 [file Table_1.docx]

**Supplementary methods**

| **Variable** | **BCAMS: 2004** | **CCACH: 2014** | **SCVBH: 2019** |
| --- | --- | --- | --- |
| **Questionnaire** |  |  |  |
| Household income  (categorical variable: poor, middle, and high) | Annual family income in ten thousand Yuan and the number of family members were collected to calculate the per capita annual household income.  The poverty threshold of Beijing in 2003 was 290 Yuan/month. Poverty-income ratio (PIR) was derived by dividing per capita annual household income by the established poverty threshold, and PIR <1.0, 1.0–<3.0, and ≥ 3.0 were defined as poor, middle, and high, respectively. | The per capita annual household income in ten thousand Yuan was collected by questionnaire.  The poverty threshold of Beijing in 2013 was 580 Yuan/month. Poverty-income ratio (PIR) was derived by dividing per capita annual household income by the established poverty threshold, and PIR <1.0, 1.0–<3.0, and ≥ 3.0 were defined as poor, middle, and high, respectively. | The per capita annual family income in ten thousand Yuan was collected as categorical variables: <2, 2-<5, 5-<12, 12-<25, 25-<50, and ≥50.  The poverty threshold of Beijing in 2018 was 1000 Yuan/month.  The per capita annual family income <2, 2-<25, and ≥25 were defined as poor, middle, and high, respectively. |
| Parental educational level  (binary variable: below college, college and above) | Father/mother education level was collected as categorical variables: under primary school, after primary school, after junior middle school, after senior middle school, after junior college, after university, and after graduate or above. | Father/mother education level was collected as categorical variables: after junior middle school, after senior middle school, after junior college, after university, and graduate or above. | Same as that in 2014. |
| Parental weight status  (categorical variable: normal, overweight, and obesity) | Parental body weight and height were collected by questionnaires. Then parental BMI was calculated as weight (kg) divided by square of height (m^2^). Parental weight status was defined as normal (<24 kg/m^2^), overweight (24 - <28 kg/m^2^), and obesity (>28 kg/m^2^). | Same as that in 2004. | Same as that in 2004. |
| Parental history of cardiovascular disease  (binary variable: yes or no) | Presence of parental CVD history including stroke, coronary heart disease, diabetes, and hypertension were collected by questionnaires.  Individuals with any presence of history of above-mentioned diseases were defined as having parental history of cardiovascular disease. | Same as that in 2004. | Same as that in 2004. |
| Passive smoking  (binary variable: yes or no) | Parental smoking status was asked by the question “Do you now smoke cigarettes?”  Individuals whose parents was a current smoker was defined as having passive smoking exposure. | Same as that in 2004. | Passive smoking information was collected by the question “During the last 7 days, how often did you stay in a room where someone else smoke for more than 15 minutes?”  Individuals who had stay in a room where someone else smoke for more than 15 minutes was defined as having passive smoking status. |
| Sedentary behavior  (binary variable: <2 h or ≥2 h) | Daily time spent on TV and computer were collected as sedentary time. | Daily time spent on screen-based leisure activities (e.g., television, computer, mobile phone) were collected as sedentary time. | Same as that in 2014. |
| Sleep duration  (binary variable: adequate or not) | Daily sleep duration in hours were collected by questionnaire.  Individuals that slept 9 to 12 h/day (6-12 years) or 8 to 10 h/day (13-18 years) were considered to have an adequate sleep duration. | Same as that in 2004. | Bedtime and wake-up time were collected by questionnaire, then the sleep duration was calculated. |
| Premature  (binary variable: yes or no) | Mother’s gestation weeks were collected using questionnaire, and premature was < 37 completed weeks of gestation | Same as that in 2004. | Same as that in 2004. |
| Sexual maturity status  (binary variable: yes or no) | Q: Have you ever had spermatorrhea (boy) / menstruation (girl)? | Same as that in 2004. | Same as that in 2004. |
| Smoking status  (binary variable: yes or no) | Q: In the last month, have you smoked one whole cigarette?  A: Tried; Never; Yes. | Same as that in 2004. | Same as that in 2004. |
| Dietary intake  (categorical variable: poor, middle, and ideal) | Q: How often do you eat (bean-curd or related, sea food/fish/ aquatic product, vegetable, fruit, dairy- products, fried food/western-style fast food, soft-drink/sugary beverage) food?  A: everyday; 3-5 times per week; 1-2 times per day; 1 time per 2 weeks; seldom or never. | Same as that in 2004. | Same as that in 2004. |
| Physical activity  (categorical variable: poor, middle, and ideal) | Q: How often do you do exercise?  A: daily outdoor activity for 30 mins; over 3 times per week and over 30 mins every time; over 1 time per week and over 30 mins per time; over 1 time per 2 weeks and over 30 mins per time; seldom or never.  The time spend going school by bicycle was collected, and then added to the time spend doing exercise. | Q: How many days do you do vigorous-intensity activities in a week?  How much time do you spend doing vigorous-intensity activities on a typical day? | Same as that in 2014. |
| **Anthropometric measure** |  |  |  |
| Body mass index | Height was measured using wall-mounted stadiometer without shoes.  Weight was measured using beam scales with light indoor clothing. | Same as that in 2004 | Same as that in 2004 |
| Blood pressure | Mercury sphygmomanometer | Oscillometric device (OMRON HEM-7012 Omron, Kyoto, Japan) | Oscillometric device (OMRON HBP-1300, Omron, Kyoto, Japan) |
| Fat mass percentage | Bioelectrical impedance analysis (TBF-300A; TANITA) | Hologic Discovery (A, W and Wi) fan-beam densitometers (Hologic, Bedford, Massachusetts, USA). | Bioelectrical impedance analysis （H-Key350, Sihaihuacheng, Beijing, China） |
| **Laboratory assay** |  |  |  |
| Total cholesterol | Finger capillary blood was determined by testing device (Accutrend GCT, Roche Diagnostics, Mannheim, Germany). | Venous blood was determined by an Olympus AU640 automatic chemistry analyzer (Olympus, Tokyo, Japan). | Venous blood was determined by an Hitachi 7080 biochemistry autoanalyzer (Hitachi, Tokyo, Japan). |
| Fasting blood glucose | Finger capillary blood were determined by testing device (Accutrend GCT, Roche Diagnostics, Mannheim, Germany). | Venous blood was determined by an Olympus AU640 automatic chemistry analyzer (Olympus, Tokyo, Japan). | Venous blood was determined by an Hitachi 7080 biochemistry autoanalyzer (Hitachi, Tokyo, Japan). |
